# Photosynthetic Competence of the Marine Aerobic Anoxygenic Phototrophic Bacterium *Roseobacter* sp. under Organic Substrate Limitation

**DOI:** 10.1264/jsme2.ME13130

**Published:** 2014-02-04

**Authors:** Yuki Sato-Takabe, Koji Hamasaki, Koji Suzuki

**Affiliations:** 1 Graduate School of Environmental Science, Hokkaido University, Sapporo, Hokkaido 060–0810, Japan; 2 Center for Marine Environmental Studies, Ehime University, Matsuyama, Ehime 790–8577, Japan; 3 Atmosphere and Ocean Research Institute, the University of Tokyo, Kashiwa, Chiba 277–8564, Japan; 4 Faculty of Environmental Earth Science, Hokkaido University, Sapporo, Hokkaido 060–810, Japan

**Keywords:** Aerobic anoxygenic phototrophic bacteria, *Roseobacter*, bacteriochlorophyll *a*, variable fluorescence, photoheterotrophy

## Abstract

This paper describes the photosynthetic response of a *Roseobacter* strain of marine aerobic anoxygenic phototrophic bacteria to an organic substrate limitation. In batch cultures, higher values of the spheroidenone/bacteriochlorophyll *a* ratio were observed under substrate-deficient conditions. Interestingly, the maximum photochemical quantum efficiencies of the photosystem under substrate-deficient conditions using blue or green excitation were significantly higher than those under substrate-replete conditions. These results indicate that spheroidenone, which can absorb green light, may play an important role in their photosynthesis as a light-harvesting antenna pigment, and the photosynthetic competence of the *Roseobacter* strain can increase in an organic substrate-deficient environment.

Aerobic anoxygenic phototrophic bacteria (AAnPB), which contain the pigment bacteriochlorophyll (BChl) *a*, have been discovered from aerobic marine environments since the late 1970s (13). AAnPB are photoheterotrophic organisms, which rely on both phototrophy and heterotrophy for the acquisition of energy, placing them somewhere in the middle of the continuum between pure photoautotrophic and heterotrophic organisms (4). AAnPB are widely distributed and their spatiotemporal changes are large in the upper oceans. Based on the ecophysiological characteristics of AAnPB, it has been hypothesized that the photoheterotrophy of AAnPB could be beneficial in oligotrophic open oceans (8–11). Suyama *et al.* (16) examined the production of photosynthetic apparatus in freshwater AAnPB in relation to the availability of carbon and light: they found that the viability of the cells in a carbon-deficient medium under aerobic and light conditions was higher than that of cells under aerobic and dark conditions, possibly due to their ATP production of cells through photosynthetic energy conversion. However, little is known about the differences in photosynthetic activity of marine AAnPB under organic substrate-replete and substrate-deficient conditions. In this study, using variable BChl *a* fluorometry, we examined the temporal changes in the maximum photochemical quantum efficiency (*F*_v_/*F*_m_) and functional absorption cross-section (σ) of the photosystem for a coastal marine AAnPB strain (OBYS 0001) belonging to the *Roseobacter* clade under both high and low organic substrate conditions. The variable BChl *a* fluorescence technique is useful for estimating the photophysiology of natural AAnPB or their isolated strains because it is non-destructive, non-invasive, rapid, sensitive and archived in real-time (see 3, 5, 6, 8, 12). In this study, cell abundance and the BChl *a* level were examined concomitantly. It is well known that the *Roseobacter* clade of AAnPB is ubiquitous in the sea (1, 17). Recently, Sato-Takabe *et al.* (12) found that the *Roseobacter* OBYS 001 strain can capture green light more efficiently than *Erythrobacter longus* for photosynthesis and that this behavior can become a survival strategy in the bacterium’s habitat. However, the response of the photosynthetic competence of *Roseobacter* to different organic substrate conditions remains poorly understood. Therefore, the results of this study could be beneficial for understanding the photophysiological response of AAnPB under organic substrate limitation.

The *Roseobacter* sp. strain OBYS 0001, isolated from Otsuchi Bay, Japan (12), was grown in 1 × and 1/100 × ZoBell 2216E medium with a salinity value of 33 (for each duplicate, the data were calculated using averages of these samples) in 2.8-L polycarbonate Erlenmeyer flasks without aeration. The 1 × ZoBell 2216E medium (substrate-replete media) contained (per liter of natural seawater) 5 g Bacto Peptone (Becton, Dickinson and Company) and 1 g Bacto Yeast Extract (Becton, Dickinson and Company), and 1/100 × ZoBell 2216E medium (substrate-deficient media) was 100 times diluted 1 × ZoBell 2216E medium. The pre-cultures were grown in 1 × ZoBell 2216E medium with a salinity value of 33 using 250-mL polycarbonate Erlenmeyer flasks without aeration, and incubated until the stationary phase. We inoculated the new medium with stationary-phase cells. Then, incubation was performed at 20°C under light and dark cycles (12 h : 12 h). Light intensity from a fluorescent lamp (FL20SS BRN/18; TOSHIBA) was set to approximately 10 μmol photons m^−2^ s^−1^ because the OBYS 0001 strain was obtained from turbid waters at a depth of 1 m. The time-course samples, which were collected daily at the end of the dark period, were filtered onto Nuclepore black polycarbonate membrane filters (0.2 μm in pore size) under a gentle vacuum (≤0.025 MPa). Cells were counted using epifluorescence microscopy (Olympus CKX-41N-FI) with the SYBR Gold staining method (14). A minimum of 10 randomly selected fields were examined, covering at least 300 bacterial cells. Triplicate samples were collected for pigment analysis. Pigments were analyzed with high-performance liquid chromatography (HPLC) according to Sato-Takabe *et al.* (12). The time-course samples for pigment analysis were collected daily at the end of the dark period. Spheroidenone/BChl *a* ratios were estimated from the peak areas of spheroidenone at 482 nm and BChl *a* at 780 nm. Variable fluorescence was measured for triplicate samples using the Satlantic FIRe (Fluorescence Induction and Relaxation) system, which contains blue (455 nm with 60 nm bandwidth) and green excitation (540 nm with 60 nm bandwidth) light-emitting diodes (LEDs). The FIRe protocol involved a strong flash of saturating blue and green, initiating an increase in fluorescence *in vivo* from the initial value (*F*_0_) to the maximum value (*F*_m_) in a single turnover flash. A strong short pulse with a duration of 80 μs (*i.e.*, a single turnover flash) induced transient changes in BChl *a* fluorescence emission at 880 nm. Before measurements, samples were dark-adapted for 30 min to open the reaction centers. Triplicate samples were measured with 10 iterations per sample. Then, filtrates of samples that had been passed through a 0.2 μm filter were used as a blank for the sample signals. Raw data were collected following the manufacturer’s protocol and processed with the MATLAB-based program fireworx, developed by Dr. Audrey B. Barnett (Dalhousie Univ.) to obtain *F*_v_/*F*_m_ and σ, which are the maximum photochemical quantum efficiency and functional absorption cross-section of the bacterial photosynthetic complexes, respectively. The excitation spectrum for BChl *a* emission in OBYS 0001 referred to Sato-Takabe *et al.* (12) for estimating the action spectrum of photosynthesis.

Cell abundance of OBYS 0001 in 1/100× ZoBell medium varied from 2 × 10^7^ to 1 × 10^8^ cells mL^−1^, while abundance ranged from 2 × 10^7^ to 6 × 10^8^ cells mL^−1^ in undiluted medium (Fig. 1A). As a result, the growth curves in both conditions clearly exhibited different patterns (Fig. 1A). These results indicate that the growth of cells in diluted medium was suppressed by the deficiency in organic substrates. The time course of BChl *a* concentrations in OBYS 0001 also exhibited different patterns between the two conditions (Fig. 1B). The BChl *a* concentrations of OBYS 0001 (Fig. 1B) under substrate-deficient conditions were maintained at a constant level (1.3 to 4.9 ng mL^−1^), while those under substrate-replete conditions gradually increased with time (1.5 to 99 ng mL^−1^). Changes over time in the cellular BChl *a* content of OBYS 0001 also exhibited different patterns between the two conditions (Fig. 1C). The cellular BChl *a* contents ranged from 34.2 to 80.6 ag per cell for OBYS 0001 under substrate-deficient conditions and from 83.3 to 196 ag per cell for OBYS 0001 under substrate-replete conditions. Under substrate-replete conditions, the cellular BChl *a* content exhibited a minimum on Day 6, which corresponded to the end of the exponential growth phase, and the highest cellular BChl *a* content was observed at the end of the experiment (*i.e.*, Day 14), showing the high production of BChl *a* in the stationary growth phase (Fig. 1C). These results are consistent with those of Sato-Takabe *et al.* (12). In contrast, under substrate-deficient conditions, the cellular BChl *a* content remained almost constant. These results indicate that pigment production under substrate-deficient conditions was suppressed because of the lack of organic substrates. Single-turnover flash saturation profiles of variable BChl *a* fluorescence using blue excitation revealed that the functional absorption cross-sections (σ) of the photosystem of OBYS 0001 were not distinct between the two conditions (Fig. 2A). However, green excitation produced different patterns between the two conditions. Green excitation yielded significantly higher σ values than did blue excitation under both substrate conditions (Fig. 2A and 2B, p <0.05, Mann-Whitney two-tailed *U*-test). This pattern suggests that OBYS 0001 can utilize green light more efficiently than blue light for photosynthesis (also see 12). The reason for the dissimilarity between the two excitations is uncertain, but it might be due to differences in the concentration of spheroidenone, which can play a major role in energy transfer processes in light-harvesting 1 (LH1)–reaction center complexes in AAnPB that belong to the *Roseobacter* clade (6, 15). The spheroidenone/BChl *a* ratio for OBYS 0001 was significantly higher under substrate-deficient condition than under substrate-replete conditions (Fig. 3; p <0.05, Mann-Whitney two-tailed *U*-test). These results indicate that spheroidenone may play a more important role as a light-harvesting pigment in AAnPB photosynthesis under substrate-deficient conditions than under substrate-replete conditions. Noteworthy, under substrate-deficient conditions, the maximum photochemical quantum efficiencies (*F*_v_/*F*_m_) of the photosystem for OBYS 0001 using blue or green excitation gradually increased with time (Fig. 2C and 2D). In contrast, those of OBYS 0001 under substrate-replete conditions changed little during the experiment (Fig. 2C and 2D). This bacterial pattern under substrate-replete conditions was consistent with the results of Sato-Takabe *et al.* (12). After Day 4, the *F*_v_/*F*_m_ values under substrate-deficient conditions became higher than those under substrate-replete conditions (Fig. 2C and 2D). These results indicate that AAnPB can enhance their photosynthetic activity under substrate-deficient conditions without any changes in cellular BChl *a* concentration (Fig. 1C). The activity of the bacterial reaction center (*i.e.*, *F*_v_/*F*_m_) might be up-regulated by respiration inhibition under organic substrate-deficient conditions, because the respiration chain of purple bacteria including *Roseobacter* is connected to their photosystem and provides electrons to the reaction center (2). However, the variable fluorescence technique with a single turnover flash used in this study can estimate the quantum efficiency of photochemistry (*F*_v_/*F*_m_) and the functional absorption cross section (σ) of the photosystem (see 6, 7). Since the single turnover flash (ca. 80 μs) causes a single reduction of the primary acceptor, Q_A_, and the respiratory chain (cytochrome *b/c*_1_ complex) is connected downstream (*i.e.*, ubiquione pool) of the photosynthetic electron transport (2), it would not be necessary to consider the connection between the photosystem and the respiratory chain, at least to interpret the fluorescence parameters. In conclusion, we found for the first time that under substrate-deficient conditions, spheroidenone, which can absorb green light, could play an important role in photosynthesis as a light-harvesting antenna pigment, and *F*_v_/*F*_m_ values significantly increased.

## Figures and Tables

**Fig. 1:**
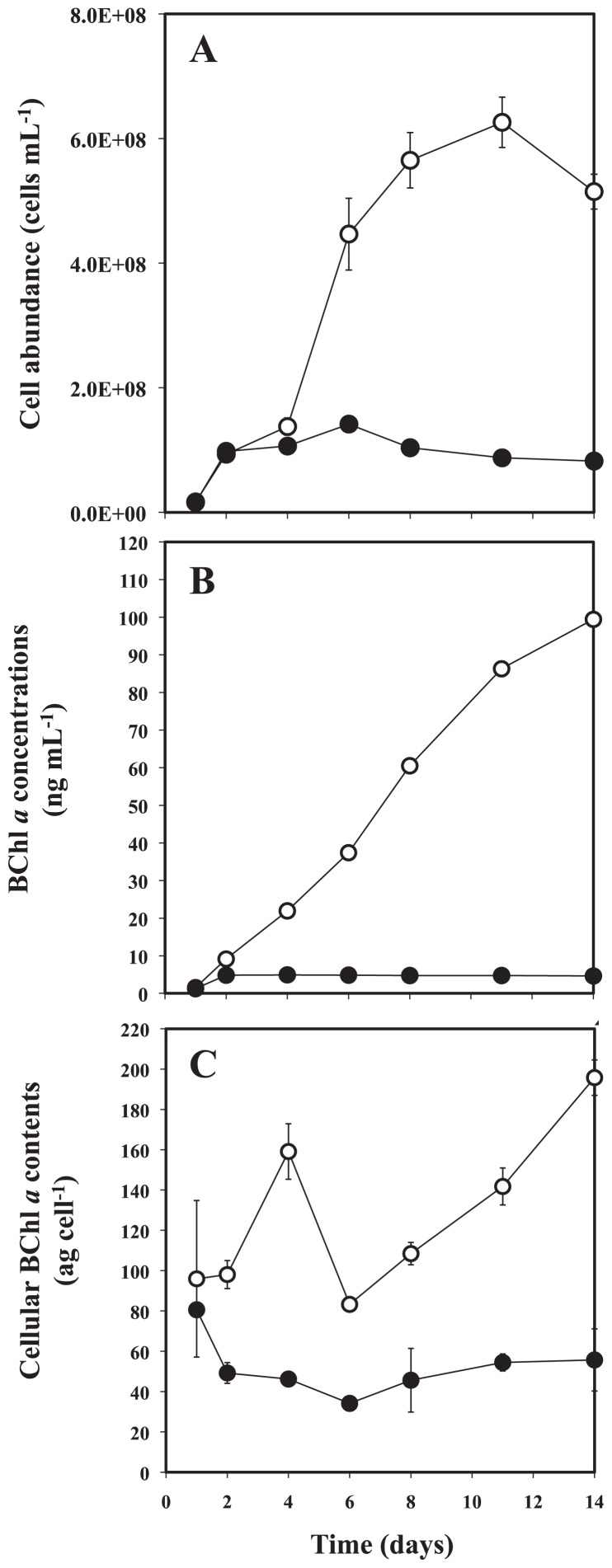
Changes over time of cell abundance (A), BChl *a* concentrations (B) and cellular BChl *a* contents (C) of strain OBYS 0001 in 1/100× (●) and 1× ZoBell 2216E medium (○). Error bar represents standard error of the mean.

**Fig. 2:**
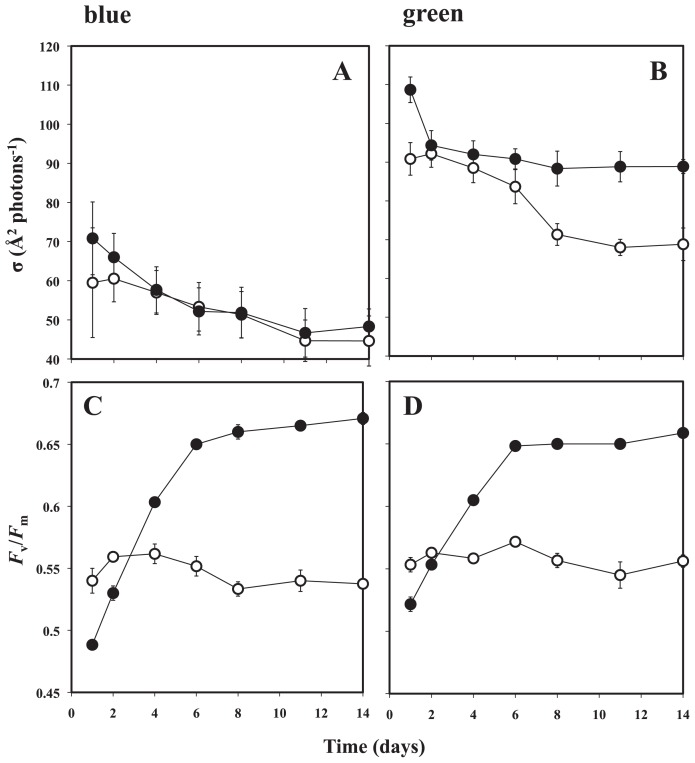
Under substrate-deficient (●) or substrate-replete conditions (○), changes over time in the photochemical quantum efficiency (*F*_v_/*F*_m_) of the photosystem for OYBS 0001 as estimated from (A) blue or (B) green excitation and the functional absorption cross-section (σ) of the photosystem using (C) blue or (D) green excitation. Error bar represents standard error of the mean.

**Fig. 3:**
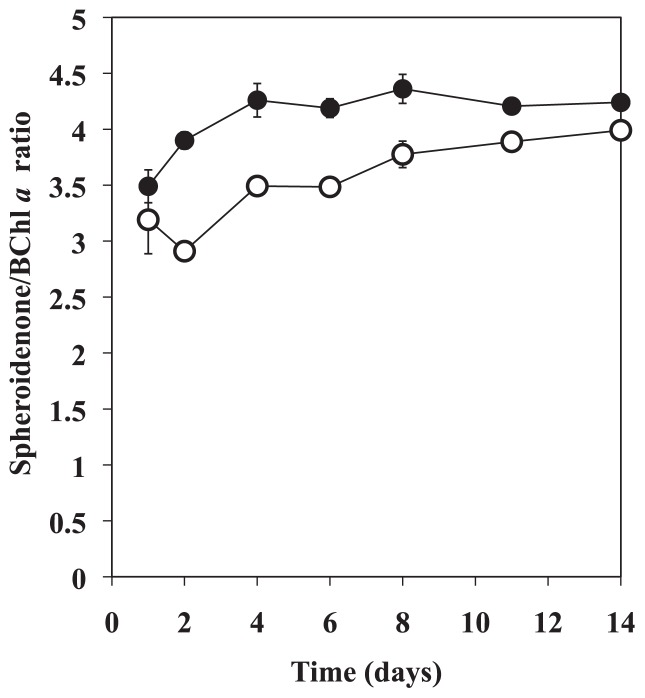
Under substrate-deficient (●) or substrate-replete conditions (○), changes over time in spheroidenone (at 482 nm) to the BChl *a* (at 780 nm) ratio for OYBS 0001 as estimated from HPLC analysis. Error bar represents standard error of the mean.
